# Draft Genome Sequence of a Thermophilic Desulfurization Bacterium, *Geobacillus thermoglucosidasius* Strain W-2

**DOI:** 10.1128/genomeA.00793-16

**Published:** 2016-08-04

**Authors:** Lin Zhu, Mingchang Li, Shuyi Guo, Wei Wang

**Affiliations:** a Key Laboratory of Molecular Microbiology and Technology, Ministry of Education, TEDA Institute of Biological Sciences and Biotechnology, Nankai University, TEDA, Tianjin, People’s Republic of China; b Tianjin Key Laboratory of Microbial Functional Genomics, TEDA, Tianjin, People’s Republic of China

## Abstract

*Geobacillus thermoglucosidasius* strain W-2 is a thermophilic bacterium isolated from a deep-subsurface oil reservoir in northern China, which is capable of degrading organosulfur compounds. Here, we report the draft genome sequence of *G. thermoglucosidasius* strain W-2, which may help to elucidate the genetic basis of biodegradation of organosulfur pollutants under heated conditions.

## GENOME ANNOUNCEMENT

*Geobacillus thermoglucosidasius* strain W-2 was isolated from a deep-subsurface oil reservoir in northern China, which can degrade organosulfur compounds. Sulfur presenting in fuels leads to SO_2_ emission during combustion, which causes not only serious air pollution, but also metal catalysts poisoning. Benzothiophene (BT) and dibenzothiophene (DBT) are the most widely studied heterocyclic sulfur compounds with respect to its susceptibility to microbial desulfurization (1, 2). To date, two major DBT biodesulfurization pathways (“Kodama” and “4S”) have been widely characterized (3–5), and a BT biodesulfurization pathway has been newly identified in a *Gordonia terrae* strain C-6 (6). In addition, alkanesulfonates are the major alkyl sulfur-containing compounds and the desulfonation mechanism has been investigated (7). However, the previously reported pathways for degrading BT, DBT and alkanesulfonates were all found in mesophilic bacteria. We report the draft genome sequence of strain W-2, which may provide further insights into genetic information of a thermophilic mechanism of biodesulfurization.

The genome sequencing of strain W-2 was carried out using the Illumina HiSeq 2500 platform at the Majorbio Bio-Pharm Technology Co., Ltd. (Shanghai, China), and Illumina paired-end (PE) libraries were constructed. *de novo* assembly was performed by using SOAPdenovo (version 2.04) and GapCloser (version 1.12). The final genome draft of strain W-2 contains 51 contigs, with a total size of 3,894,555 bp and an average G+C content of 43.34%. The average contig length was 76,664 bp, with the largest contig being 560,848 bp. Gene prediction and annotation were performed as described previously (8). As a result, 3,935 protein-encoding genes, one rRNA operon, and 76 tRNA genes for all 20 amino acids were predicted.

Genes encoding for three putative alkanesulfonate monooxygenases, seven putative sulfonate ABC transporters, and two putative sulfate permeases were identified in the draft genome. They were hypothesized to be responsible for organosulfur compounds degradation (9–11). In addition, we also found some genes that encode nitronate monooxygenases, which catalyze oxidative denitrification of nitroalkanes to carbonyl compounds and nitrites (12). It may be the first time that the two groups of enzymes responsible for biodesulfurization and biodenitrification pathways have been found in thermophilic bacteria. The functions and mechanisms of the two biodegradation pathways in strain W-2 are under investigation. As thermophilic enzymes offer major biotechnological advantages over mesophilic enzymes (13), the draft genome of strain W-2 may provide an excellent platform for further improvement of this organism for bioremediation and other biotechnological applications at elevated temperature.

### Accession number(s).

The whole-genome shotgun project of *G. thermoglucosidasius* strain W-2 has been deposited at DDBJ/EMBL/GenBank under the accession number LXMA00000000. The version described in this paper is the first version, LXMA01000000.
